# Rural Poor Economies and Foreign Investors: An Opportunity or a Risk?

**DOI:** 10.1371/journal.pone.0114703

**Published:** 2014-12-15

**Authors:** Angelo Antoci, Paolo Russu, Elisa Ticci

**Affiliations:** 1 Department of Economics and Business-CRENoS, University of Sassari, Sassari, Italy; 2 Department of Economics and Statistics, University of Siena, Siena, Italy; University of the Basque Country, Spain

## Abstract

In the current age of commercial and financial openness, remote and poor local economies are becoming increasingly exposed to inflows of external capital. The new investors - enjoying lower credit constraints than local dwellers - might play a propulsive role in local development. At the same time, inflows of external capital can have negative impacts on local natural resource-dependent activities. We analyze a two-sector model where both sectors damage the environment, but only that of domestic producers relies on natural resources. We assess under which conditions the coexistence of the two sectors is compatible with sustainability, defined as convergence to a stationary state characterized by a positive stock of the natural resource. Moreover, we find that capital inflows can be stimulated by an increase in the pollution intensity of incoming activities, but also in the pollution intensity of the domestic sector; in both cases, capital inflows generate environmental degradation and a decrease in welfare for the local population. Finally, we show that a reduction in the cost of capital for external investors and the consequent capital inflows have the effect to increase wages, local investments and welfare of the local populations only if the environmental impact of the external sector is relatively low with respect to that of local activities. Otherwise, an unexpected scenario characterized by a reduction in domestic capital accumulation and the impoverishment of local agents can occur.

## Introduction

In poor economies, foreign direct investments (FDI) are seen by policy makers as a possible solution to tackle the scarcity of domestic capitals and to escape a poverty trap of low investments, low growth and the perpetuation of poverty. The arrival of external investors is usually considered to be beneficial for economic expansion and for the diversification of local economies. Many governments offer significant inducements to attract FDI. [1] calculates that 2078 out of 2267 national policy changes, introduced around the world between 1992 and 2005, were favorable to FDI. A vast part of the economic literature regards FDI inflows as positive drivers of economic development in recipient countries since they can generate spill-over effects on local firms through diffusion of technology and more advanced management practices ([2], [3], [4], [5], [6], [7], [8]) or since they may foster the creation of employment, development of infrastructure, expansion of the tax base, and the collection of fiscal revenues ([9], [10], [11]). Other studies focus on the conditions required to produce these positive effects ([12], [13], [14], [15], [16], [17], [18], [19], [20], [21], [22], [23]). Less attention has been dedicated to the potentially negative impacts of external capital investments notwithstanding the findings of some authors that in certain developing countries foreign investments can harm or crowd-out local firms or have negative effects on economic growth in the short term ([24], [25], [26], [27], [28], [29], [30], [31], [32]).

Overall, the empirical evidence on the impact of FDI on local development and local firms, is still mixed ([20], [33]). At the same time, the ongoing and numerous episodes of environmental-related protests by local populations against large foreign or national investment projects all over the world suggest that negative interactions between natural resource dependent activities and large investors may be both significant and severe. The information gathered in the Environmental Justice Atlas (EJ Atlas), for istance is emblematic. The EJ Atlas is a global database created within the Environmental Justice Organisations, Liabilities and Trade (EJOLT) project that monitors and collects data on environmental conflicts, namely mobilizations by local communities or social movements against particular economic activities, in which environmental impacts are a key element of their grievances. Though the platform does not cover all geographic areas, it has already recorded 1122 cases of ecological distribution conflicts. Increasing material demand (land, water, mineral ores, biomass), waste creation and industrial production seem to feed a growing climate of protests by communities trying to defend their livelihoods from damaging environmental impacts. The databases collects stories of extractive projects, mega dams, large transport infrastructures, toxic waste, dump sites, energy and power plants, large-scale land acquisitions, pollution due to manufacturing. China provides some of the most symbolic examples of rural communities harmed by the arrival of new manufacturing firms. In February 2012, Chinese premier Wen Jiabao said that: “ Water pollution is mainly resulting from industrial and sewage waste water and is now in very serious situation.” (reported in Greenpeace International, 2012). Li Yang, Vice-President of the Chinese Academy of Social Sciences, said in February 2013 that “ China's real economic growth rate would only be around 5%, if economic losses caused by ecological degradation and environmental damage are subtracted from the overall GDP” (http://news.xinhuanet.com/english/indepth/2013-02/28/c_132312932.htm). Recent evidence suggests that global extraction of natural resources from ecosystem has increased consistently over the last forty years despite a considerable decline in the material intensity of production [34]; the dematerialization of production in rich countries is also explained by growing material imports from abroad. [35] using a global input–output database (Eora MRIO database) find that developed countries import water embodied in goods from the rest of the world to reduce pressure on domestic water resources. Similarly, the time series analysis of material requirements for consumption (material footprint) of 186 countries by [36] suggests that as advanced economies become richer, they are able to increase their material consumption while at the same time reducing their extraction of domestic materials through international trade. The indirect impact of this transition is a growing absorption of environmentally intensive activities by lower income countries.

All these factors suggest that the development process of today's less developed countries cannot be viewed in isolation from the environmental pressures exerted by external forces. This paper contributes to the debate on the relationship between external investments, poverty reduction and sustainability by discussing how this link is affected by the environmental attributes of the recipient economy, in the form of initial endowments of natural capital and environmental carrying capacity, and the pollution intensity of economic activities. We assume that foreign and local firms are heterogeneous. First, they are characterized by different production functions, that is, they belong to different sectors and they are not competitors. Only the local sector relies on natural resources. Second, the law of motion of physical capital differs between the two sectors due to capital market imperfections. We reckon that this is an important extension as many rural economic activities in developing countries are characterized by dependence on environmental resources and barriers to credit markets ([37], [38]). At the same time, for the sake of clarity and analytical convenience, we exclude some of the positive channels of transmission that have been studied by FDI literature such as inward or forward linkages, knowledge spillovers, and increased access to international market networks. However, some potential positive impacts of FDI are embodied in the model. Capital inflows create new labor opportunities and raise labor demand. This, in turn, increases wage labor remuneration in the external sectors and the resulting growth in the revenues of local workers expands their savings and their capacity to invest. In other words, we assume that in the economy under study, where economic agents can invest only to the extent they save, revenues generated from FDI can foster the domestic accumulation of physical capital.

## Model and Methods

Let us consider a simplified economy where prices are exogenous and there are three factors of production: labor, a renewable natural resource and physical capital. The agents are divided in two population groups: “ External Investors” (I-agents) and “ Local Agents” (L-agents). The I-agents are endowed with physical capital which can be invested in the economy in question or elsewhere. We assume that they do not face credit constraints and their availability of physical capital is “ unlimited”. Therefore they will continue to invest their capital in the economy as long as the return on capital generated is higher than in other economies. I-agents also employ wage labor and undertake all their potential work - represented by a fixed amount of entrepreneurial activity - in what we call the “ external sector”. The main asset of the L-agents is labor force and they have to choose how to distribute this asset between two activities: wage work for External Investors in the external sector or direct exploitation of the natural resource. Let us say that “ local sector” denotes production of the Local Agents. Their activities can be represented by natural resource harvesting, fisheries, forestry or tourism. Given that L- and I- agents' investments in physical capital follow different mechanisms and rules, we assume that the capital market is completely segmented and that it is accessible only to the External Investors, while the Local Agents can only invest their savings.

We assume that the production functions of the two sectors satisfy the Inada conditions, are concave, increasing and homogenous of degree 1 in their inputs. The production function of the representative L-agent is given by:
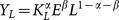
where:


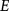
 is the stock of a free access environmental resource;


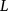
 is the amount of time the representative L-agent spends on local sector production;


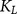
 is the physical capital accumulated by the representative L-agent;


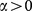
, 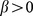, 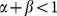 hold.

The L-agent's total amount of time is normalized to 1 and leisure is excluded, thus 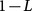 represents the L-agent's labor when employed by the representative I-agent in wage work. The production function of the representative External Investor is represented by the Cobb-Douglas function:
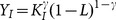
(1)where 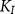 denotes the stock of physical capital invested in the economy by the representative I-agent. The I-agents choose their labor demand 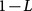 and the stock of physical capital 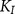 which they invest in the economy in order to maximize their profits:
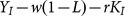
where 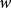 and 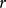 are, respectively, the wage and the interest rate, considered as exogenously determined by each I-agent. However, the wage 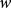 is endogenously set in the economy by the labor market equilibrium condition (we rule out the importation of labor from other economies), while 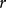 is an exogenous parameter. We assume that the 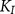 inflow is potentially unlimited. Therefore the dynamics of 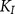 are not linked to the I-agents' savings but only to the productivity of 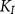 (which, in turn, depends on 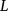 and 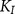). The local agents can choose the level of their savings and the allocation of their labor between the two sectors. We assume that the representative L-agent solves the following maximization problem:
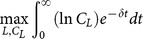
(2)subject to:
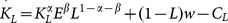
where the positive parameter 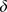 represents the discount subjective rate and 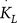 is the time derivative 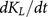 of the variable 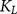. The representative local agent invests her remaining savings after financing her consumption 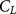 in physical capital. Her resources come from self-employment in the local sector (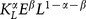) and from wage labor in the external sector (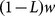). To simplify, we have assumed that the prices of the goods produced in the local and in the external sectors are both equal to unity; the wage 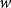 is expressed in terms of the external sector output.

We assume that both populations are made up of a continuum of identical individuals and the size of each community is equal to 1. The dynamics of 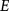 are described by a logistic function modified to take into account the impact of economic activities:
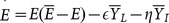




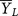
 and 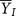 are the aggregate values of 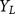 and 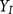 respectively and 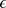 and 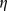 are positive parameters measuring the environmental impact caused by the aggregate production of L and I-agents respectively. The expression 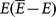 represents an inverted-U-shaped function commonly used to describe the dynamics of a renewable environmental resource without human impact. According to this, the stock 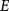 of the natural resource grows at a positive but declining rate until it reaches the maximum sustainable value 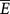; the positive parameter 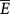 represents the carrying capacity of the environmental resource. In our model, this function is modified by adding the negative impacts caused by the production of the two sectors, which is assumed to be proportional to the aggregate production levels 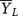 and 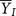.

This assumption is usual in models with economic activities depending on open-access resources (see, for example, [39], [40]) or with polluting industries ([41], [42]).

Problem (2) will be analyzed with the following restrictions on variables and parameters: 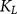, 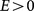; 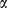, 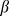, 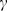, 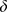, 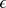, 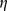, 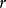, 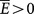; 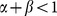.

Each economic agent considers the effect of her choices on the dynamics of 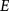 to be negligible and does not internalize it. That is, 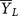 and 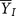 are considered to be exogenous, which implies that the evolution of 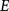 is taken as given in problem (2). As a result, the economic agents behave without taking into account of the shadow value of the natural resource and nobody invests to restore the natural capital. Working under the assumption that each typology of agents consists of a continuum of identical individuals of size 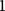, (ex post) aggregate outputs 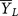 and 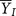 coincide with per-capita values 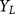 and 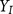, respectively.

This is obviously a stylized scenario, but it reflects some of the main differences between local and foreign firms in rural poor areas. Poor local producers usually adopt labor intensive techniques, use unpaid family work, and face strong constraints in their access to credit markets. In contrast, external investors are able to finance their activities by borrowing from the capital markets, hire workers, and adopt capital-intensive techniques. Their ability to accumulate physical capital is typically in no way comparable to that of the local population. Local and external agents also differ in terms of defensive strategies. External investors can react to a reduction in the return on physical capital by moving to other economies. Local producers, on the other hand, cannot displace their economic activities and can only defend themselves from the reduced productivity of the assets used in their activities, natural and physical capital, by modifying the allocation of their fixed amount of labor across sectors and by choosing the level of their savings under a strict budget constraint.

### Dynamics

The dynamics generated by the model are derived in S1 File by applying the *Maximum Principle* to the maximization problem (2) of the representative L-agent, under the condition of labor market equilibrium. According to the results contained in S1 File, we have that the function:
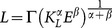
(3)where:
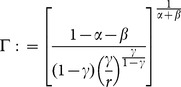
identifies the labor market equilibrium value 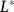 of 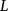 if the right side of (3) is lower than 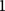; otherwise, the equilibrium value of 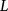 is 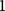, that is:
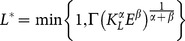
(4)


The economy is specialized in the production of the L-sector if 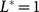. Its specialization in the production of the external sector is excluded (that is, 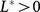 always holds). Therefore two cases can occur: the case *without specialization* (in the local sector) and the case *with specialization*. It is worth observing that the external sector never completely replaces the local sector, since the productivity of labor employed in local activities tends to infinity as the workers move away from this sector. In contrast, the economy can fully specialize in the local sector although the productivity of labor in the external sector also tends to infinity as 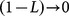. In this case, in fact, external investors withdraw their capitals from the economy and reduce 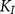, which eventually goes to zero, in that labor becomes increasingly expensive.

#### Dynamics without specialization

If 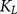 and 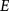 are such that 
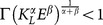
 (see (4)), then L-agents spend a positive fraction of their time endowment working in the external sector and equation (3) identifies the equilibrium value of 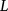. Moreover, the following proposition holds:

**Proposition 1**: *The equilibrium wage rate is constant and is given by* 
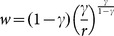
.

**Prof**: *See* *S1 File*.

The equilibrium wage rate is completely determined by the elasticity with respect to labor of production in the external sector (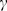) and by the capital cost (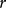). A change in any other parameter does not affect the equilibrium wage even if it leads to a variation in the equilibrium values of 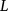, 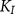 and of 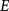. We will return later on the implications of this result.

In this context, the dynamics obtained by applying the *Maximum Principle* to problem (2) can be expressed as follows:
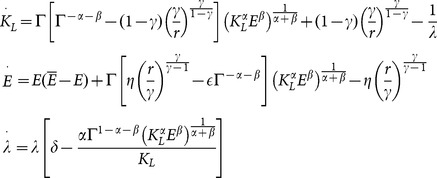
(5)where 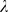 is the co-state variable associated to the state variable 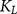, which is usually interpreted as the “ price” of 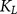.

#### Dynamics with specialization

If 
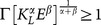
, then the L-agents spend all their time endowment working in the L-sector, that is 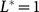, and the dynamic system obtained by applying the *Maximum Principle* to problem (2) is:
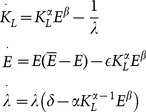
(6)


#### Stationary states of dynamics

A stationary state 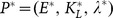 of the dynamics (5)-(6) is a solution of the system 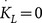, 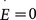, 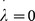. From equation 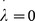, in the case without specialization (system (5)), we obtain:
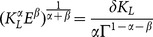
(7)


Substituting (7) in (3), we get:
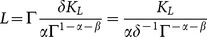
(8)


Consequently, 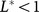 if and only if 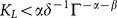. This implies that the stationary states without specialization lie below the horizontal line in the plane 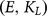:
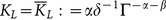
(9)


It is easy to check that, below the straight line 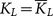, the stationary states (without specialization) are given by the intersections between the two following curves:
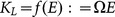
(10)

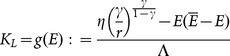
(11)where:
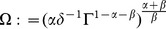
(12)

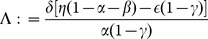
(13)


Notice that 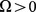 always while:
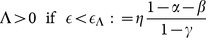
(14)


The existence and stability properties of the stationary states without specialization pivot on the sign of the parameter 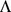 which indicates whether shifting the labor force from one sector to the other results in an increase or in a decrease of the environmental impact. In particular, according to (14), 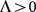 holds if the ratio between 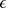 (measuring the environmental impact of the local sector) and 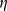 (measuring the environmental impact of the external sector) is lower than the ratio between the labor elasticities, 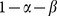 and 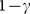, of the production functions in the local sector and in the external sector, respectively.

Above 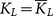, the stationary states with specialization (system (6)) are given by the intersections between the following two curves:
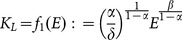
(15)

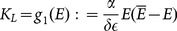
(16)


The following propositions deal with the problem of the existence and numerosity of the stationary states of the dynamics (5)–(6).

**Proposition 2**: *The dynamic system (5)–(6) admits at most four stationary states*: 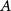 *and* 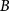 *with* 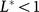, 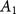 *and* 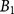 *with* 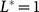.

**Proof**: *The graph of* 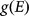 *is a parabola while the graph of* 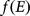 *is a straight line, consequently* 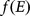 *and* 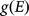 *have at most two intersections. In the same way, both* 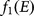 *and* 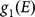 *are concave, however the difference* 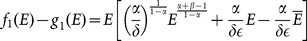 *has at most two zeros with* 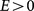.

The symbol 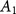 (respectively, 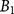) shall refer to the stationary state 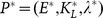 with specialization satisfying the condition 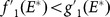 (respectively, 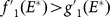); in the same way, the symbol 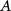 (respectively, 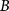) shall refer to the stationary state without specialization satisfying the condition 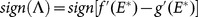 (respectively, 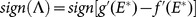).

To express the next proposition, we have to define the following threshold values:
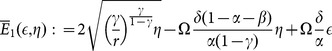
(17)

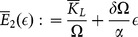
(18)

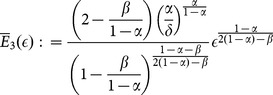
(19)

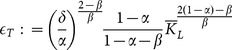
(20)

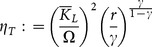
(21)


where 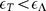 (see (14)) if:
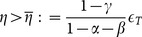
(22)


**Figure 1:**
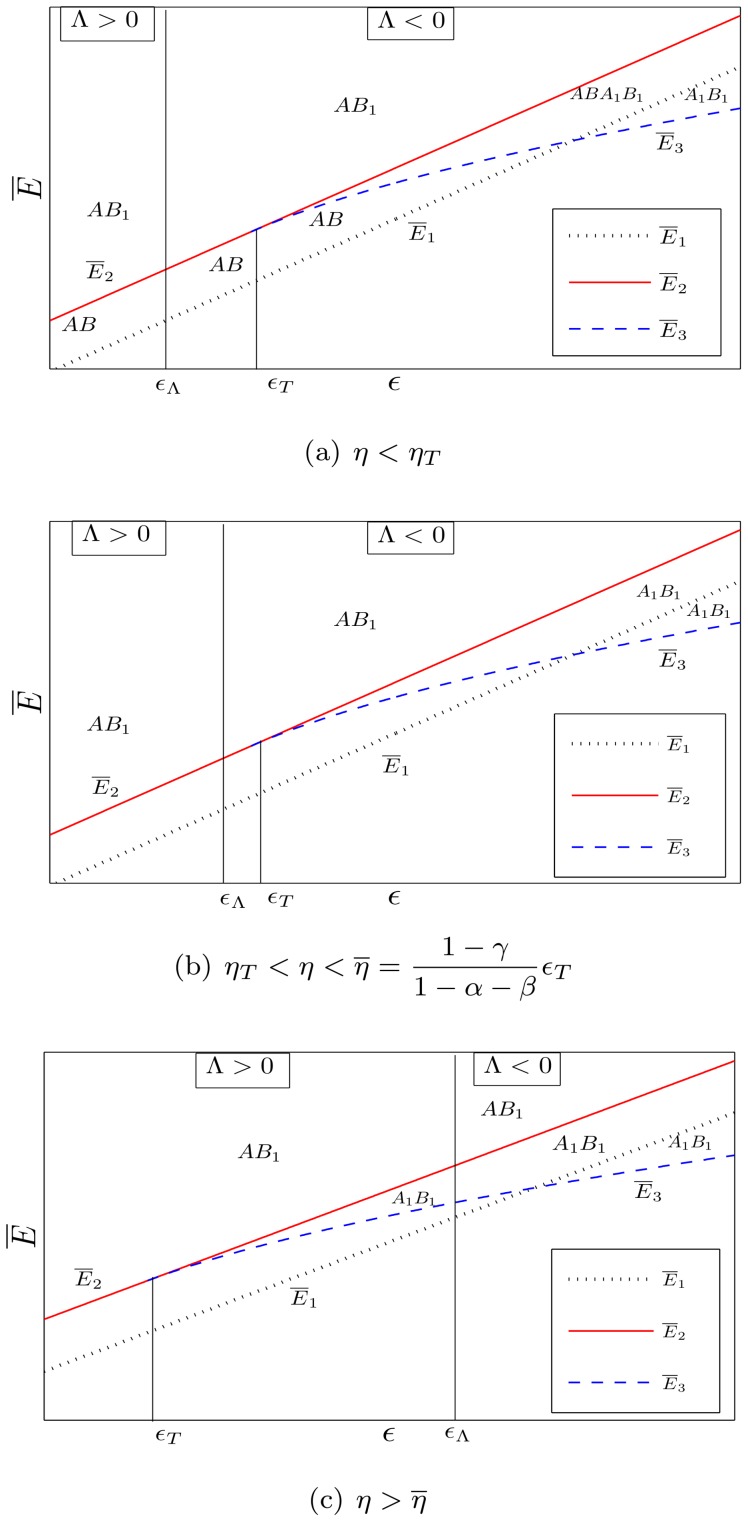
Threshold values in the plane 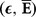 and existing stationary states.

**Proposition 3**: *The stationary states of the dynamic system (5)–(6) are (see* *Fig. 1* *)*:

1) 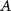, 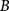, 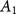, 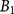 *if and only if (iff)*:
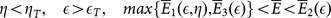



2) 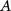 *and* 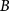 *iff*:
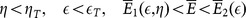

*or*

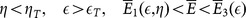



3) 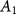 *and* 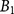 *iff*:
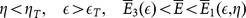

*or*

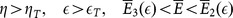



4) 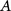 *and* 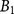 *iff*:
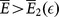



*No stationary state exists in the remaining cases.*

**Proof**: *See* *S1 File*.

In the above classification, for the sake simplicity, we do not take into account “ non robust” cases corresponding to an equality condition of parameter values (for example, the cases in which 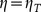, 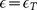 or 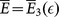).

**Figure 2:**
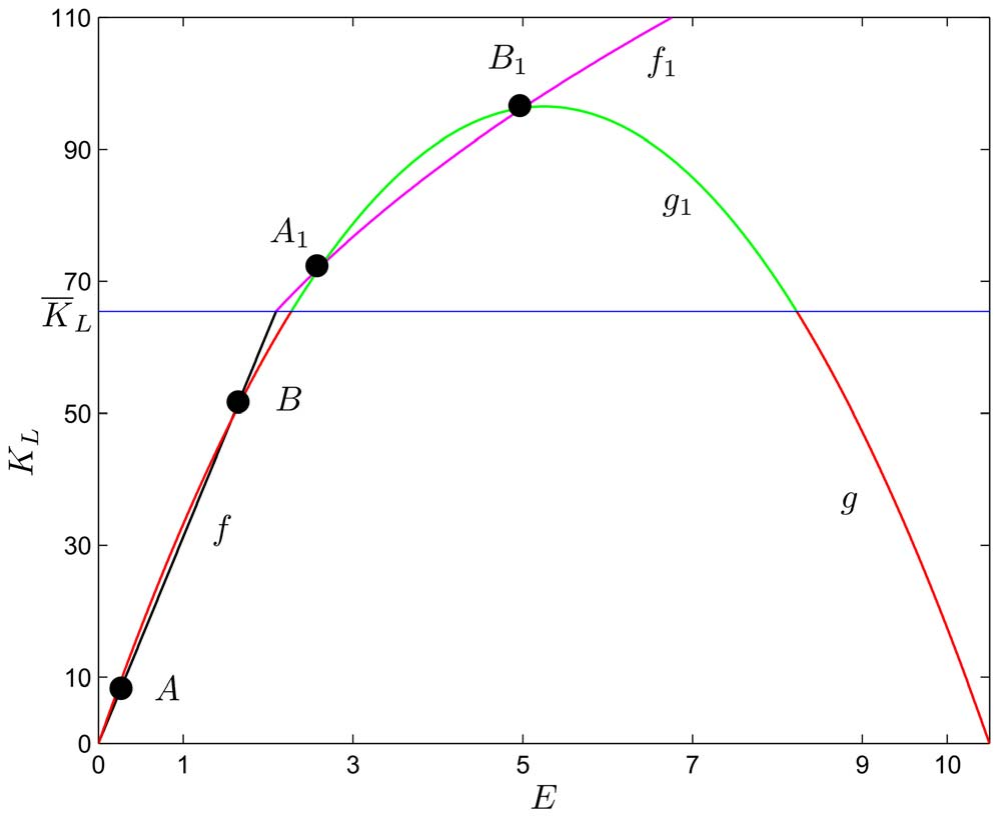
Numerical example in which four stationary states exist; the values of parameters are: 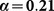, 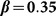, 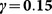, 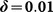, 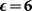, 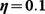, 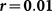, 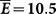.

Fig. 2 shows a numerical example in which four stationary states exist: 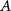, 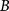, 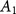 and 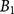.

Let 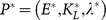 be a stationary state of the dynamics (5)–(6). The stability properties of 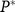 depend on the signs of the real parts of the eigenvalues associated to the Jacobian matrix 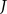 evaluated at 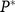. We shall say that 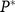 is *saddle-point stable* if 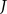 has two eigenvalues with negative real parts, i.e. if 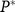 has a 2-dimensional stable manifold. As a matter of fact, under the perfect foresight assumption, when the stationary state 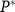 has a 2-dimensional stable manifold, if the initial values 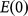 and 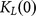 are near enough to 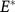 and 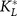, L-agents are able to fix the initial value 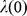 of the (jumping) co-state variable 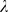 so that the growth trajectory starting from 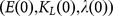 approaches 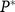. Therefore the stationary state can be reached by growth trajectories. If 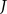 has less than two eigenvalues with negative real parts, then given the initial values 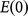 and 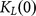, a value 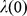 does not (generically) exist so that the growth trajectory starting from 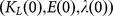 approaches the stationary state.

The following propositions concern the stability properties of the stationary states 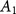 and 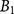, in the regime with specialization (i.e. 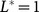), and the states 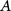 and 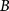, in the regime without specialization (i.e. 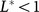). Numerical example in which four stationary states exist; the values of parameters are: 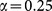, 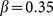, 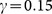, 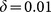, 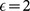, 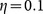, 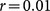, 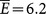.

**Proposition 4**: *In the regime with specialization, we have that: 1) If* 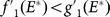 *(that is,* 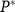 *is of the type* 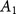), *then* 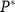 *has two eigenvalues with strictly positive real parts and one with strictly negative real part; 2) If* 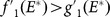 *(that is,* 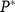 *is of the type* 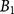), *then* 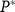 *is saddle-point stable or it has three eigenvalues with strictly positive real parts; a sufficient condition for saddle-point stability is*:
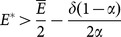



**Proof**: *See* *S1 File*.

The following proposition deals with the stability properties of the stationary states 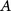 and 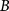 in the regime without specialization (i.e. 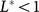).

**Proposition 5**: *In the regime without specialization, we have that: 1) If* 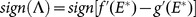 *(that is,* 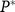 *is of the type* 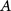), *then* 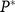 *has two eigenvalues with strictly positive real parts and one with strictly negative real part. 2) If* 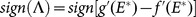 *(that is,* 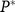 *is of the type* 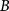), *then* 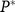 *is saddle-point stable or it has three eigenvalues with strictly positive real parts; a sufficient condition for saddle-point stability is*:
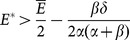



**Proof**: *See* *S1 File*.

Remember that the parameter 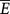 represents the carrying capacity of the environmental resource 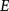 while the parameters 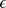 and 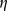 measure the environmental impact caused by the aggregate production of L-agents and I-agents, respectively. Fig. 1 shows the regions, in the plane 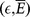, corresponding to cases 1-4 of Proposition 3.

Joining together the results concerning the existence and the stability of the stationary states, we can observe that:

1. A bistable regime (case 1 of Proposition 3, where all the stationary states 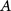, 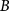, 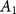, 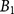 exist) can be observed only for low enough values of 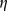 (i.e. 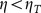) and high enough values of 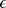 (i.e. 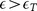), see Fig. 1(a); furthermore, the carrying capacity 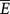 must lie between 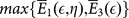 and 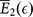. In this context, the dynamics are path dependent: if the economy starts near enough to 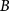 (respectively, to 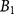), then the stationary state 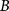 (respectively, 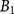) is reached.
2. A necessary condition for the existence of a stationary state of type 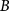 is 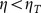 (see Fig. 1(a)). In this case, both the local and external sectors can coexist in a stationary state. If the external sector produces a devastating environmental effect (i.e. 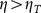), then the expansion of the external sector generates an increase in environmental degradation which, in turn, fuels a further expansion of the external sector. This self-enforcing mechanism is incompatible with the sustainable coexistence of the two sectors. Furthermore, the stationary state of type 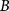 does not exist if (ceteris paribus) the carrying capacity 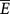 is high enough; in this a case, only the stationary states 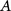 and 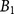 exist (see Figs. 1(a)-1(c)). The coexistence of the two sectors can be observed only if (see Fig. 1(a)) 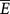 is neither “ too low” nor “ too high” (i.e. 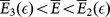).
3. When 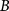 and 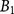 do not simultaneously exist, then at most one saddle-point stable stationary state can exist. In particular, only 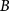 exists if the conditions expressed in case 2 of Proposition 3 hold while only 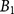 exists in the context of cases 3 and 4 of Proposition 3.

### Local agents' welfare at the stationary states

The following propositions help identify the most significant conditions that are verified in correspondence with the stationary states of dynamic system (5)–(6); the asterisk indicates the stationary state values of the variables.

**Proposition 6**: *The following conditions hold at the stationary states of the dynamic system (5)–(6)*:
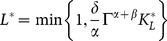
(23)

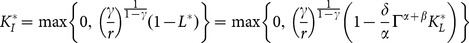
(24)

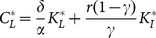
(25)


*Furthermore, in the context* 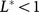 *(stationary states* 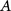 *and* 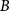), *the following condition holds*:
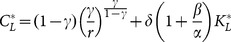
(26)


**Proof**: See S1 File.

Notice that the values of 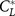 given by the formulas (25) and (26) coincide for 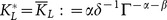.

We can observe that, according to (26), 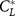 is composed by two addends: the wage rate 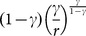 (see Proposition 1), namely the return to employment in the capitalistic sector, and 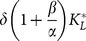 which positively depends on the physical capital accumulated by local agents at the stationary state. Variations in parameters 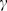 and 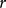, which cause an increase in the wage rate, can also rise the welfare of local agents to the extent that the possible negative impact on 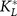 is limited. In this case, the crowding-out of the local sector is welfare-improving, otherwise the local population might experience a welfare loss even with increased wages.

Notice also that the value of 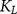, evaluated at the stationary state 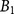, is higher than that evaluated at 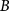 (when both 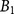 and 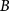 simultaneously exist) because 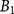 lies above the line (see (9)) 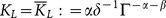 while 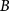 lies below it (see Fig. 2). Furthermore, also the value of 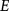 in 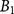 is higher than in 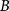; therefore, it is easy to check the following proposition:

**Proposition 7**: *When the stationary states* 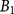 *and* 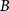 *coexist, then L-agents' welfare (measured by* 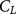), *evaluated in* 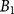, *is higher than that evaluated in* 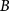.

We do not consider the stationary states 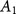 and 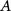 because they cannot be saddle-point stable.

Proposition 7 entails that, in a bistable regime, economies characterized by low initial values of 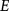 and 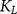 (see Fig. 2) are more likely to converge to the Pareto-dominated stationary state without specialization than richer economies, which can rely on greater values of 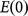 and 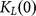 and, therefore, are located closer to the stationary state 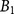.

### Comparative statics

**Figure 3:**
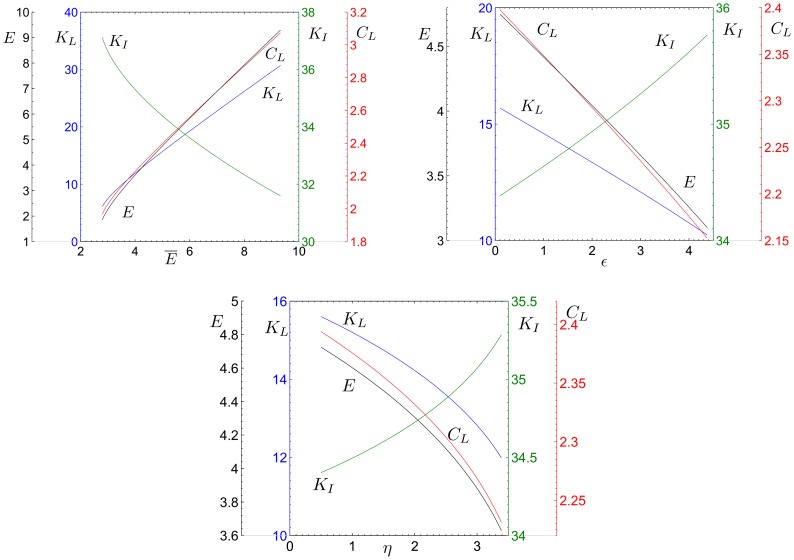
Values of 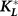, 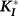, 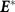, 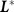, 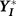 and 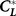 evaluated at 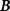, varying the parameters 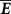, 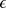 and 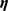; the other parameters are fixed at the values: 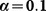, 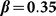, 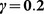, 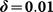, 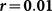.

**Figure 4:**
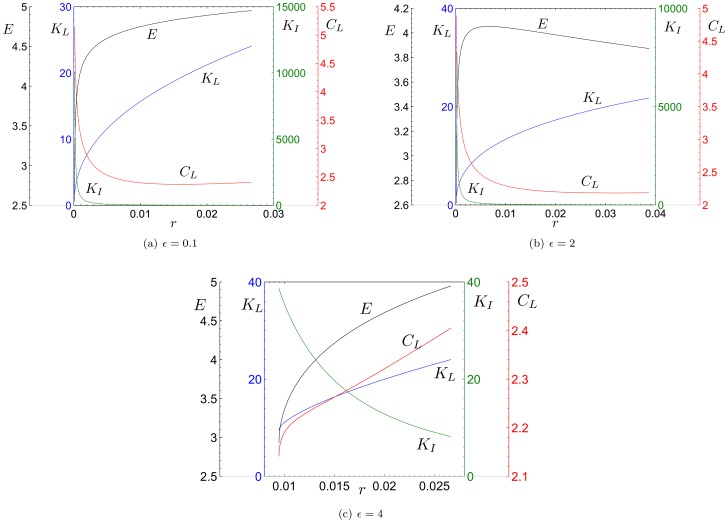
Values of 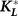, 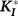, 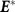, 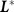, 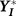 and 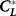 evaluated at 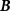, varying the parameter 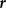; the other parameters are fixed at the values: 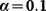, 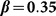, 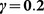, 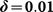, 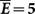.

The following propositions investigate the impact of a change in parameters (in particular, we focus our analysis on 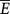, 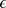, 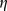, 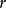) on the values of 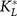, 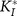, 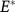, 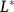, 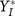 and 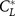 evaluated at 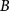, the stationary state without specialization that can be saddle-point stable. The symbols 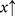 and 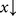 indicate, respectively, an increase and a decrease in the parameter or variable 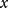. We carry out some exercises in comparative statics in 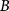 in order to assess the impact of various changes in parameters in an economy that can converge to a stationary state with two sectors, namely in a context where inflows of external capital are admissible and, in principle, do not threaten sustainability. Figs. 3 and 4 show a graphical representation of the results by some numerical exercises.

**Proposition 8**: 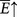 *(remember that* 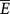 *represents the carrying capacity of the environmental resource) implies* 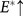, 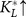, 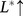, 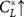 *and* 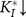


**Proof**: *See* *S1 File*.

**Proposition 9**: 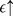 *or* 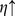 *(remember that* 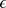 *and* 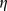 *represent, respectively, the environmental impact of the local sector and of the external one) imply* 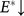, 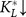, 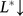, 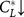 *and* 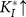


**Proof**: *See* *S1 File*.

These results show that an increase in the value of 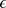 or 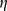 tends to stimulate a movement of labor towards the external sector, whose productive performance is not damaged by environmental degradation. The local community faces a reduction in the return to self-employed labor and is pushed towards wage employment. In this context, the expansion of external capital inflows does not help local agents. On the contrary, their welfare declines (i.e. 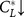). This outcome comes out even if the labor market is perfectly competitive and is not segmented, that is a context where a rise in external investor's demand for domestic labor produces a pattern of labor reallocation that should raise wages in both sectors. The introduction of environmental externalities, on the other hand, mitigates this channel of transmission resulting in an expansion of the external sector's output share with constant wages and a decrease in local agents' welfare. Indeed, as reported in Proposition 1, the equilibrium wage rate is constant and is not affected by variations in parameters 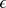 and 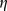.

A symmetrically opposite effect is produced by an increase in 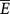, which translates into an increase in the welfare of L-agents and a reduction in the investments of I-agents. In this context, therefore, an abundance of natural capital is a blessing for local economy expansion.

The following proposition considers the effects of a change in the value of the parameter 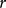, that is, a change in external agents' cost of capital investment in the economy.

**Proposition 10**: *If* 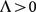 *(i.e.* 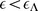 ; *see (14) and* *Fig. 1(a)* *), then* 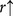 *always implies* 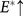 *and* 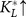. *If* 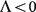 *(i.e.* 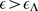), *then: 1*) 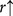 *implies* 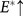 *if*:
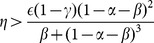
(27)


*2)* 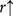 *implies* 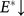 *if:*

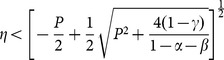

*where*:
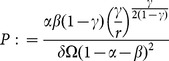



3) 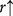 *implies* 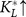 *if and only if*:
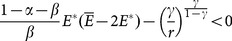
(28)


*Furthermore, (27) is a sufficient condition to have* 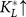.

**Proof**: *See* *S1 File*.

According to (14), condition 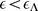 can be rewritten as 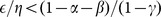, which requires (ceteris paribus) a low enough ratio between the negative environmental impact of the local sector (measured by 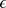) and of the external sector (measured by 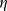).

Remember that, according to Proposition 6, 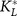 is positively correlated with 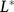 and negatively correlated with 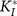; furthermore:
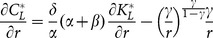
(29)


This implies that the condition 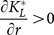 is necessary but not sufficient to obtain 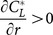. Our results (see Proposition 3 and formula (29)) predict that a reduction in 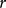 surely generates an increase in 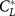 only if 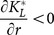; this happens when, in the context 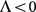 (i.e. 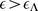), the opposite of condition (28) holds. In fact, in this case, according to formula (29), 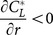 holds and so a decline in the opportunity cost of capital for the external investors leads to an increase in 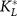 and in 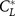. Remember that the condition 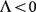 requires (ceteris paribus) a relatively low environmental impact of the external sector in comparison to that of the local activities (see (14)). In the context 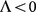, external capital inflows, which attract the labor force towards the external sector, alleviate pressure on natural resources because the local population has the opportunity to rely more on cleaner activities. In all other cases, a decline in 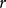 can have the effect of reducing local agents' welfare. Inflows of external capitals can still have a positive effect even when 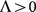, that is when external activities are relatively more polluting than local ones and their expansion is associated with a negative net impact on natural resources. Figs. 4(a) and 4(c), for instance, show two examples with 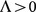 which differ only in the value of 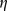. With a sufficiently low 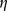, a decrease in 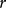 leads to a growth in 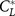 though it causes also a decline in 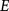. With high values of 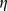, however, the opposite is true and external investments have a detrimental effect on both the local agents' welfare and the environment. This result indicates that external capital inflows, might not only fail to trigger net positive effects, but may also produce a net negative impact on local welfare even if they stimulate labor demand.

## Results and Discussion

This section gathers together and summarizes the main findings of our analysis. We have studied an economy exposed to environmental externalities and open to external investors with better access to the capital market than local producers. In this context, we find that:

1. The following three main contexts can be considered (see Figs. 1(a)–1(c)):
  - (a) If the carrying capacity (measured by the parameter 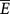) of the environment is very low, a stationary state with a positive value of the stock of the environmental resource does not exist; this implies that the economy cannot follow a path of sustainable development, that is a path along which the stock of the environmental resource is not completely exhausted.
  - (b) If the carrying capacity of the environmental resource is very high, the economy converges to a stationary state with complete specialization in the local sector (stationary state 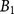).
  - (c) If the carrying capacity of the environmental resource is neither too high nor too low compared to the environmental impact of the local sector (measured by the parameter 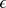), two dynamic regimes can emerge: the context in which the environmental impact of the external sector (measured by the parameter 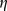) surpasses a threshold level specified in the analysis (i.e. 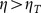) or that in which it remains below this threshold level (i.e. 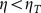):
    - (i) In the former case (illustrated in Figs. 1(b)–1(c)), the economy can only converge to a stationary state (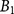) with full specialization in the local sector. Therefore, the coexistence between the local and external sectors cannot be observed in a non-transient way in that the environmental impact of the external sector is too high.
    - (ii) In the second context (represented in Fig. 1(a)), in which the intensity of the environmental impact produced by the external sector is low, at most four stationary states (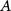, 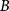, 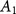 and 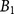) exist and at most two of them (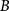 and 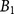) can be achieved by the economy. In this context, a bistable regime can be observed: the economy converges to the stationary state (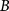) where the local and the external sectors coexist in a non transient way, or it progressively specializes in the local sector (i.e. it converges to 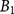) and the external sector is pushed out.
2. In the bistable scenarios described above, the selection between the two reachable stationary states depends on initial conditions (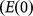 and 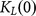). Economies starting with deteriorated environment (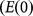 low) and low stock of domestic physical capital (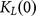 low) tend to attract external investments and to converge to a stationary state involving the coexistence of local and external activities (the state 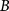). Economies characterized by higher initial endowments of domestic capital and natural resources are more likely to completely specialize in the local sector production (that is, to converge to the state 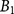). The equilibrium value of local agents' welfare is higher in these latter economies than in those with poorer initial environmental or capital endowments which converge to a stationary state of economic diversification (i.e. with the coexistence of the external and domestic sectors). These findings are consistent with the notion that if nature's bounty is sufficiently large and well-preserved and the economy starts from a sizeable initial endowment of physical capital, the mobilization of domestic resources is preferable to a development path fed by external capitals.
3. If the economy converges to the stationary state characterized by the non transient and sustainable coexistence of both sectors (the stationary state 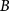), exogenous variations in the parameters' values may affect the equilibrium values of the variables in the model. In particular:
  - (a) An (exogenous) rise in the intensity of environmental pressure of productive activities (that is, an exogenous rise in the values of parameters 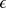 and 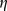) always leads to an increase in external investment, and to a reduction in the stock of the natural resource and local agents' capital accumulation and consumption, though the equilibrium wage rate is not affected. Indeed, if the interest rate and physical capital elasticity of the external sectors do not vary, international capital mobility results in constant equilibrium wages. In other words, a rise in the environmental impact of economic activities leads to a reduction in natural capital pushing part of the labor force to the external sector, depressing the accumulation capacity of local producers and, consequently, encouraging external capital inflows. The same effects are generated by an exogenous reduction in the carrying capacity of the natural resource (that is, a reduction in the value of the parameter 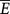).
  - (b) A decline in external investors' cost of capital in the economy (represented by the parameter 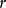) generates an increase in the external investment and a rise in the equilibrium wage. However, local agents' welfare (measured by the consumption level 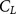) increases only if the external sector has a sufficiently low negative impact on the natural resource compared with the environmental pressure exerted by the local sector. Otherwise, scenarios characterized by an increase in external capital inflows accompanied by decreasing welfare for the local population cannot be ruled out. More precisely, if the incoming activities are very polluting, the model can generate a paradox of increased wages and rise in external capital flows associated with declining local welfare. In this case, the environmental impact of external capitals is so devasting that the new external investments generate an increase in labor demand and the remuneration of wage labor which are offset by their negative impact on labor productivity in the local sector. Incoming capital flows crowd out the local sector and do not produce a sufficient expansion of labor demand.
4. Local population's consumption when the economy converges to a state with the coexistence of the two sectors (the stationary state 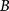) can be either positively or negatively correlated with the dynamics of natural capital and physical capital invested by external agents. The sign of this relationship depends on the factor causing the change. If the trigger factor is a variation in the carrying capacity of the environment or in the degree of environmental pressure generated by either local or external production activities, local welfare is positively correlated with the stock of natural capital and negatively correlated with the stock of external investments. If the trigger factor is a change in the cost of capital for external investors (measured by the parameter 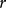, which may be also interpreted as the external agents' opportunity cost of the investment in the economy in question), then the local population's welfare is positively associated with inward external capitals only if the external sector does not pollute too heavily. In other words, an improvement in the investment climate conditions for external investors (that is, a reduction in 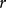) is not necessarily beneficial for the host economy. In fact, it is only certain to have a positive impact on local agents' welfare if the pollution intensity of the activities in which incoming capital inflows are employed is relatively lower than that of local activities. In this scenario, the external capitals feed an increase in wages which in turn helps the capital accumulation of local producers. The arrival of external capitals promotes the mobilization of local accumulation potential. In all other cases, instead, capital inflows may reduce local agents' welfare.

An improved investment climate is one of the main objectives of most local and national governments all over the world. The promotion of incentives and of opportunities for firms to invest and to create jobs is regarded as a crucial strategy in order to stimulate economic growth and reduce poverty. The economic doctrine has underscored the need both to mobilize domestic resources and to attract external capitals and several international organizations have suggested measures for promoting domestic investments and improving investment climate. The World Development Report 2005, for example, points out that investment climate improvements are driving factors in boosting economic expansion and combating poverty, recommending the promotion of domestic investments and support of small and rural firms [43]. In poor economies, inflows of external investments are seen by policy makers as the main solution to tackle the scarcity of domestic capitals and to escape a poverty trap of low investments - low growth - perpetual poverty. The expansion of new and non resource-based activities, prompted by the arrival of external investors, is considered to be the way forward in terms of economic expansion and diversification of the local economy. Many countries, therefore, have focused their efforts on reforms and inducements aimed at promoting big modern companies, which are usually financed by external capitals. The model proposed has enabled us to discuss the effects of economic diversification in an economy dependent on free access natural resources. The model shows that the conditions for economic development and poverty reduction become stricter when environmental dynamics are included in the debate on the interaction between local and external producers. In particular, the increasing exposure of local economies to external forces and investments can cause negative consequences. This occurs when incoming actors invest in contaminating industries and enter economies characterized by high dependence on primary activities and, consequently, by an acute vulnerability to environmental degradation or to exclusion from the use of natural resources.

However, these conclusions, need to be evaluated taking into account that positive externalities and backward or forward linkages between the two sectors are excluded from our model. The inclusion of these channels of interaction between the two sectors may, in fact, limit or downsize the results obtained by this model. Our objective, in any case, was to focus on factors that tend to be neglected in the discussion of investment incentives, namely the environmental externalities of human activities and agents' heterogeneity in terms of their vulnerability to depletion of natural resources and access to capital markets.

## Supporting Information

S1 File **Proofs of Propositions.**
(PDF)
