# Supplementary material for: Rural Poor Economies and Foreign Investors: An Opportunity or a Risk?
Source: PLoS One. 2014 Dec 15;9(12):e114703. doi: 10.1371/journal.pone.0114703 (PMC4266523; doi:10.1371/journal.pone.0114703)
Supplement: S1 File — Proofs of Propositions. (PDF) [file pone.0114703.s001.pdf]

Supporting Information to:

Rural poor economies and foreign investors: an opportunity or a risk?

Angelo Antoci[1], Paolo Russu[2,*], Elisa Ticci[3]

1,2 Department of Economics and Business-CRENoS, University of Sassari, Sassari, Italy

3 Department of Economics and Statistics, University of Siena, Siena, Italy

***E-mail: russu@uniss.it**

Proof of Proposition 1: The dynamics generated by the model are obtained by applying the *Maximum Principle* to the maximization problem of the representative L-agent, under the equilibrium condition in the labor market. The current value Hamiltonian function associated to problem (2) is (see [1]):

$$H = \ln C_L + \lambda \left[K_L^{\alpha} E^{\beta} L^{1-\alpha-\beta} + (1-L)w - C_L \right]$$

where λ is the co-state variable associated to K_L. By applying the Maximum Principle, the dynamics of the economy are described by the equations:

$$\dot{K}_L = \frac{\partial H}{\partial \lambda} = K_L^{\alpha} E^{\beta} L^{1-\alpha-\beta} + (1-L)w - C_L \tag{S.1}$$

$$\dot{\lambda} = \delta \lambda - \frac{\partial H}{\partial K_L} = \lambda \left[\delta - \alpha K_L^{\alpha-1} E^{\beta} L^{1-\alpha-\beta} \right] \tag{S.2}$$

with the constraint:

$$\dot{E} = E(\overline{E} - E) - \epsilon \overline{Y}_L - \eta \overline{Y}_I \tag{S.3}$$

where C_L and L satisfy the following conditions[1]:

$$\frac{\partial H}{\partial C_L} = \frac{1}{C_L} - \lambda = 0 \tag{S.4}$$

$$\frac{\partial H}{\partial L} = \lambda \left[(1-\alpha-\beta) K_L^{\alpha} E^{\beta} L^{-\alpha-\beta} - w \right] = 0, \text{ i.e. } w = (1-\alpha-\beta) K_L^{\alpha} E^{\beta} L^{-\alpha-\beta} \tag{S.5}$$

At the same time, the representative I-agent chooses the level of labor demand $1-L$ and physical capital

[1]Notice that, in our context, $C_L > 0$ and $1 > L > 0$ always hold.

K_I employed in the external production in order to maximize her profit function:

$$\Pi_I = K_I^{\gamma}(1-L)^{1-\gamma} - w(1-L) - rK_I \tag{S.6}$$

This gives rise to the following first order conditions:

$$\frac{\partial \Pi_I}{\partial (1-L)} = (1-\gamma)K_I^{\gamma}(1-L)^{-\gamma} - w = 0 \tag{S.7}$$

$$\frac{\partial \Pi_I}{\partial K_I} = \gamma K_I^{\gamma-1}(1-L)^{1-\gamma} - r = 0 \tag{S.8}$$

The labor market is perfectly competitive and wages are flexible. I- and L- agents take w as given, but the wage rate and labor allocation between the two sectors continue to change until the labor demand is equal to labor supply. The labor market equilibrium condition is given by:

$$(1-\gamma)K_I^{\gamma}(1-L)^{-\gamma} = (1-\alpha-\beta)\, K_L^{\alpha}E^{\beta}L^{-\alpha-\beta} \tag{S.9}$$

By equation (S.8) we have:

$$K_I = \left(\frac{\gamma}{r}\right)^{\frac{1}{1-\gamma}}(1-L) \tag{S.10}$$

and substituting K_I in (S.9) we obtain:

$$L = \Gamma\left(K_L^{\alpha}E^{\beta}\right)^{\frac{1}{\alpha+\beta}} \tag{S.11}$$

where:

$$\Gamma := \left[\frac{1-\alpha-\beta}{(1-\gamma)\left(\frac{\gamma}{r}\right)^{\frac{\gamma}{1-\gamma}}}\right]^{\frac{1}{\alpha+\beta}}$$

Function (S.11) identifies the labor market equilibrium value L^* of L if the right side of (S.11) is lower than 1; otherwise, the equilibrium value of L is 1, that is:

$$L^* = \min\left\{1,\, \Gamma\left(K_L^{\alpha}E^{\beta}\right)^{\frac{1}{\alpha+\beta}}\right\} \tag{S.12}$$

The economy is specialized in the production of the L-sector if $L^* = 1$. Note that condition (S.5) excludes the specialization in the production of the external sector (i.e. $L^* > 0$ always). Therefore two cases are

distinguished, the case *without specialization* (in the local sector) and the case *with specialization.*

If $\Gamma \left(K_L^\alpha E^\beta\right)^{\frac{1}{\alpha+\beta}} < 1$, then L-agents spend a positive fraction of their time endowment working in the external sector and condition (S.11) identifies the equilibrium value of L. The equilibrium wage rate is constant and is given by $w = (1-\gamma)\left(\frac{\gamma}{r}\right)^{\frac{\gamma}{1-\gamma}}$ (Proposition 1); to prove such result, let us substitute (S.11) in (S.5) obtaining:

$$\begin{aligned} w &= (1-\alpha-\beta)\, K_L^\alpha E^\beta L^{-\alpha-\beta} = \\ &= (1-\alpha-\beta)\, K_L^\alpha E^\beta \left[\Gamma \left(K_L^\alpha E^\beta\right)^{\frac{1}{\alpha+\beta}}\right]^{-\alpha-\beta} = \\ &= (1-\gamma)\left(\frac{\gamma}{r}\right)^{\frac{\gamma}{1-\gamma}} \end{aligned}$$

In such a context, the dynamic system (S.1)-(S.3) can be written in the form (5).

If $\Gamma \left[K_L^\alpha E^\beta\right]^{\frac{1}{\alpha+\beta}} \geq 1$, then L-agents spend all their time endowment working in the L-sector, that is $L^* = 1$, and the dynamic system (S.1)-(S.3) can be written in the form (6).

Proof of Proposition 3: Notice that: a) $f(E)$ and $f_1(E)$ do not depend on the parameter $\overline{E}$ (see (10) and (15)); b) if the value of the parameter $\overline{E}$ increases, the graph of $g(E)$ moves up (in the plane (E, K_L)) in the case $\Lambda < 0$ and moves down in the case $\Lambda > 0$; c) if the value of the parameter $\overline{E}$ increases, the graph of $g_1(E)$ moves up.

The classification given in this proposition, based on the values of the parameters $\overline{E}$, ϵ, η and represented in the plane $(\epsilon, \overline{E})$ (see Figure 1), can be easily checked considering that the thresholds values defined in formulas (17)-(21) are characterized by the following properties:

1) *given ϵ and η, the function $\overline{E}_1(\epsilon, \eta)$ (see (17)) indicates the value of the parameter $\overline{E}$ such that the curves $f(E)$ and $g(E)$ are tangent*;
   proof: Remember that $f(E) = \Omega E$ and $g(E) = \Phi - \dfrac{E(\overline{E}-E)}{\Lambda}$, where $\Phi = \dfrac{\eta(\frac{\gamma}{r})^{\frac{\gamma}{1-\gamma}}}{\Lambda}$. Consider the quadratic equation $f(E) - g(E) = 0$, whose solutions are:

$$E_{1,2} = \frac{1}{2}(\overline{E} + \Omega\Lambda) \pm \frac{1}{2}\sqrt{(\overline{E} + \Omega\Lambda)^2 - \Phi} \tag{S.13}$$

   Equating the discriminant of (S.13) to zero, we get $\overline{E} = 2\sqrt{\Phi} - \Omega\Lambda$; replacing Φ, Ω by (12) and Λ by (13), we obtain $\overline{E}_1(\epsilon, \eta) = \overline{E}$.

2) *given ϵ, the function $\overline{E}_2(\epsilon)$ (see (18)) indicates the value of the parameter $\overline{E}$ such that the curves*

$f(E)$ and $g(E)$ have an intersection point along the horizontal line $K_L = \overline{K}_L$ ($\overline{K}_L$ is defined in (9))[2];

proof: Posing $\overline{K}_L = f(E)$, we get $E = \dfrac{\overline{K}_L}{\Omega}$ that replaced in $\overline{K}_L = g(E)$ leads to $\overline{K}_L = \Phi - \dfrac{\overline{K}_L}{\Omega\Lambda}(\overline{E} - \dfrac{\overline{K}_L}{\Omega})$, that is $\overline{E} = \dfrac{\overline{K}_L}{\Omega} - \Omega\Lambda + \Phi\dfrac{\Omega\Lambda}{\overline{K}_L}$. Remembering the values of Φ, Λ and $\overline{K}_L$, we obtain $\overline{E}_2(\epsilon) = \overline{E}$;

3) *given ϵ, the function $\overline{E}_3(\epsilon)$ (see (19)) indicates the value of the parameter $\overline{E}$ such that the curves $f_1(E)$ and $g_1(E)$ are tangent*;

proof: Let us rewrite the functions $f_1(E)$ and $g_1(E)$ as follows:

$$K_L = f_1(E) = AE^{\rho} \tag{S.14}$$

$$K_L = g_1(E) = \frac{B}{\epsilon}E(\overline{E} - E) \tag{S.15}$$

where $A = \left(\frac{\alpha}{\delta}\right)^{\frac{1}{1-\alpha}}$, $B = \dfrac{\alpha}{\delta}$ and $\rho = \dfrac{\beta}{1-\alpha}$. By solving of system:

$$\begin{cases} f_1(E) = g_1(E) \\ f_1'(E) = g_1'(E) \end{cases} \tag{S.16}$$

where $f_1'(E)$ and $g_1'(E)$ represent the derivatives with respect to E of $f_1(E)$ and $g_1(E)$, we get

$$E_T = \left(\epsilon\frac{A}{B}(1-\rho)\right)^{\frac{1}{2-\rho}} \tag{S.17}$$

and

$$\overline{E}_T = \frac{2-\rho}{1-\rho}E_T \tag{S.18}$$

where $\overline{E}_T$ and E_T indicate respectively, the values of $\overline{E}$ and E in the tangency point.

Substituting (S.17) and the values of A, B and ρ in (S.18) we obtain $\overline{E}_3(\epsilon) = \overline{E}_T$

4) *the tangency point between the curves $f_1(E)$ and $g_1(E)$ lies above the horizontal line $K_L = \overline{K}_L$ if and only if the condition $\epsilon > \epsilon_T$ (see (20)) is satisfied*;

[2]Notice that, in such a case, the intersection point between $f(E)$ and $g(E)$ coincides with that between $f_1(E)$ and $g_1(E)$.

proof: Substituting the equation (S.17) in (S.14) and solving for ϵ the following system:

$$\begin{cases} K_L = A\left(\epsilon\dfrac{A}{B}(1-\rho)\right)^{\frac{\rho}{2-\rho}} \\ K_L = \overline{K}_L \end{cases} \tag{S.19}$$

we obtain $\epsilon = \epsilon_T$. From the system (S.19), we can see that ϵ is positively correlated to K_L, then $\epsilon > \epsilon_T$ implies $K_L > \overline{K}_L$.

5) *the tangency point between the curves $f(E)$ and $g(E)$ lies below the horizontal line $K_L = \overline{K}_L$ if and only if the condition $\eta < \eta_T$ (see (21)) is satisfied*;

proof: To prove this item we follow the steps:

a) Posing $f'(E) = g'(E)$ we obtain:

$$\overline{E} = 2E - \Lambda\Omega \tag{S.20}$$

b) By substituting $\overline{E} = \overline{E}_1(\epsilon, \eta)$ (remember that $\overline{E}_1(\epsilon, \eta)$ indicates the value of the parameter $\overline{E}$ such that the curves $f(E)$ and $g(E)$ are tangent, see point 1) and $E = \dfrac{K_L}{\Omega}$ (obtained from the equation $K_L = f(E)$) in equation (S.20), it becomes:

$$2\sqrt{\left(\frac{\gamma}{r}\right)^{\frac{\gamma}{1-\gamma}}\eta} - \Omega\frac{\delta(1-\alpha-\beta)}{\alpha(1-\gamma)}\eta + \Omega\frac{\delta}{\alpha}\epsilon = 2\frac{K_L}{\Omega} - \Lambda\Omega \tag{S.21}$$

c) By straightforward calculations, equation (S.21) can be written as:

$$\sqrt{\left(\frac{\gamma}{r}\right)^{\frac{\gamma}{1-\gamma}}\eta} = \frac{K_L}{\Omega} \tag{S.22}$$

Substituting $K_L = \overline{K}_L$ in equation (S.22) we get $\eta = \eta_T$, furthermore given the positive correlation between K_L and η, we have that $\eta < \eta_T$ implies $K_L < \overline{K}_L$;

6) $\epsilon_\Lambda < \epsilon_T$ *holds, (remember we have that $\Lambda > 0$ iff $\epsilon < \epsilon_\Lambda$, see (14)) if and only if $\eta < \overline{\eta} := \dfrac{1-\gamma}{1-\alpha-\beta}\epsilon_T$, where $\overline{\eta} > \eta_T$ always holds*;

proof: To prove the first part of the above item, note that being $\epsilon_\Lambda = \dfrac{1-\alpha-\beta}{1-\gamma}\eta$, then $\epsilon_\Lambda < \epsilon_T$ if $\eta < \dfrac{1-\gamma}{1-\alpha-\beta}\epsilon_T$.

Now to prove that $\overline{\eta} > \eta_T$, we write this inequality as follows:

$$\frac{1-\gamma}{1-\alpha-\beta}\epsilon_T > \eta_T \tag{S.23}$$

then, substituting the values of ϵ_T and η_T (given by formulas (20) and (21) respectively) and noting that by (12), $\Omega = (\overline{K}_L\Gamma)^{\frac{\alpha+\beta}{\beta}}$ holds, the inequality (S.23) becomes:

$$\overline{K}_L > \overline{K}_L\left(\frac{1-\alpha-\beta}{1-\alpha}\right)^{\frac{\beta}{2-\beta}} \tag{S.24}$$

which, being $\dfrac{1-\alpha-\beta}{1-\alpha} < 1$, is always satisfied;

7) *the graphs of $\overline{E}_1(\epsilon,\eta)$ and $\overline{E}_2(\epsilon)$, in the plane $(\epsilon,\overline{E})$, are two parallel straight lines; they coincide for $\eta = \eta_T$ while $\overline{E}_1(\epsilon,\eta)$ lies below $\overline{E}_2(\epsilon)$ for $\eta \neq \eta_T$;*

proof: Let us write equations (17) and (18) as follows:

$$\overline{E}_1(\epsilon,\eta) = 2\sqrt{\left(\frac{\gamma}{r}\right)^{\frac{\gamma}{1-\gamma}}\eta} - \Omega\frac{\delta(1-\alpha-\beta)}{\alpha(1-\gamma)}\eta + \Omega\frac{\delta}{\alpha}\epsilon \tag{S.25}$$

$$\overline{E}_2(\epsilon) = \frac{\overline{K}_L}{\Omega} + \Omega\frac{\delta}{\alpha}\epsilon \tag{S.26}$$

where it is easy to see that they have, in the plane $(\epsilon,\overline{E})$, the same slope $\dfrac{\Omega\delta}{\alpha}$.

To prove the second part of the above item, it is sufficient to show that the function:

$$V(\eta) := \overline{E}_2(\epsilon) - \overline{E}_1(\epsilon,\eta) = \frac{\overline{K}_L}{\Omega} + \Omega\frac{\delta(1-\alpha-\beta)}{\alpha(1-\gamma)}\eta - 2\sqrt{\left(\frac{\gamma}{r}\right)^{\frac{\gamma}{1-\gamma}}\eta} \tag{S.27}$$

has the following properties:

a) $V(\eta_T) = 0$;

b) it presents a minimum at $\eta = \eta_T$.

The propriety *a)* can be easily checked by noting that, by (21):

$$\Omega\frac{\delta(1-\alpha-\beta)}{\alpha(1-\gamma)}\eta_T = \sqrt{\left(\frac{\gamma}{r}\right)^{\frac{\gamma}{1-\gamma}}\eta_T} = \frac{\overline{K}_L}{\Omega}$$

To prove the propriety *b)*, it is sufficient to check that the derivative of the function $V(\eta)$ with

respect to η, evaluated at $\eta = \eta_T$, that is:

$$\left(\frac{dV(\eta)}{d\eta}\right)_{\eta=\eta_T} = \Omega\left(\frac{\delta(1-\alpha-\beta)}{\alpha(1-\gamma)} - \left(\frac{\gamma}{r}\right)^{\frac{\gamma}{1-\gamma}}\frac{1}{\overline{K}_L}\right)$$

is equal to zero, and that the second order derivative $\left(\frac{d^2V(\eta)}{d\eta^2}\right)_{\eta=\eta_T} = \frac{\sqrt{\left(\frac{\gamma}{r}\right)^{\frac{\gamma}{1-\gamma}}}}{\eta_T^2}$ is strictly positive;

8) *the function $\overline{E}_3(\epsilon)$ is strictly concave in ϵ, its graph lies below the straight line $\overline{E}_2(\epsilon)$ and is always tangent to it for $\epsilon = \epsilon_T$.*

proof: Let us rewrite equation (19) as follows:

$$\overline{E}_3(\epsilon) = A\epsilon^{\phi} \tag{S.28}$$

Differentiating twice $\overline{E}_3(\epsilon)$ with respect to ϵ we obtain $\overline{E}_3''(\epsilon) = \phi(\phi-1)\epsilon^{\phi-2}$, consequently, being $\phi - 1 < 0$, the function (S.28)is strictly concave. To prove the second part of the above item, is sufficient to show that $\overline{E}_2(\epsilon)$ is the tangent line to the graph $\overline{E}_3(\epsilon)$ at $\epsilon = \epsilon_T$, that is $\overline{E}_2'(\epsilon) = \overline{E}_3'(\epsilon)$ for $\epsilon = \epsilon_T$. In fact, we have $\frac{\delta\Omega}{\alpha} = A\phi\epsilon^{\phi-1}$, that is $\epsilon = \left(\frac{\alpha A\phi}{\delta\Omega}\right)^{\frac{1}{1-\phi}} = \epsilon_T$.

Proof of Proposition 4: This proof and the following one are built on [1]. The Jacobian matrix $J_1(P^*)$, evaluated at a stationary state with specialization $P^* = (E^*, K^*, \lambda^*)$, can be expressed as follows:

$$J_1(P^*) = \begin{pmatrix} \delta & \frac{\beta\delta}{\alpha}\frac{K_L^*}{E^*} & \frac{1}{(\lambda^*)^2} \\ -\delta\epsilon & \overline{E} - 2E^* - \frac{\beta\delta\epsilon}{\alpha}\frac{K_L^*}{E^*} & 0 \\ \delta(1-\alpha)\frac{\lambda^*}{K_L^*} & -\beta\delta\frac{\lambda^*}{E^*} & 0 \end{pmatrix}$$

The eingenvalues of $J_1(P^*)$ are the roots of the following characteristic polynomial:

$$P_1(z) = z^3 - tr(J_1)z^2 + Mz - |J_1|$$

where:

$$tr(J_1) = \overline{E} + \delta - 2E^* - \frac{\beta\delta\epsilon}{\alpha}\frac{K_L^*}{E^*}$$

$$M = \delta(\overline{E} - 2E^*) - \frac{\delta^2(1-\alpha)}{\alpha}$$

$$|J_1| = \frac{\delta}{\lambda^*}\left[\frac{\beta\delta\epsilon}{\alpha}\frac{1}{E^*} - (1-\alpha)\frac{\overline{E} - 2E^*}{K_L^*}\right] \tag{S.29}$$

It easy to check that the determinant $|J_1|$ can be expressed as follows:

$$|J_1| = \frac{\epsilon\delta^2(1-\alpha)}{\alpha\lambda^* K^*}\left[f_1'(E^*) - g_1'(E^*)\right]$$

where f_1' and g_1' are the derivatives of f_1 and g_1 evaluated at E^*. Therefore, $|J_1| \gtreqless 0$ for $f_1'(E^*) - g_1'(E^*) \gtreqless 0$. Since, at the stationary state A_1, the condition $f_1'(E^*) < g_1'(E^*)$ holds, A_1 is either a saddle with two eingenvalues with strictly positive real parts or a sink; however, A_1 cannot be a sink in that, by (S.29), $|J_1| > 0$ implies $tr(J_1) > 0$.

At the stationary state B_1, the condition $f_1'(E^*) > g_1'(E^*)$ holds; therefore B_1 is either a saddle with two eingenvalues with strictly negative real parts or a source. [1] finds that $M < 0$, i.e. $E^* > \frac{1}{2}\left(\overline{E} - \frac{\delta(1-\alpha)}{\alpha}\right)$, is a sufficient condition for the saddle-point stability of B_1. This completes the proof.

Proof of Proposition 5: The Jacobian matrix $J(P^*)$, evaluated at a stationary state without specialization $P^* = (E^*, K^*, \lambda^*)$, can be expressed as follows:

$$J(P^*) = \begin{pmatrix} \delta & \frac{\beta\delta\Omega}{\alpha} & \frac{1}{(\lambda^*)^2} \\ \frac{\alpha\Lambda}{\alpha+\beta} & \overline{E} - 2E^* + \frac{\beta\Lambda\Omega}{\alpha+\beta} & 0 \\ \frac{\beta\delta}{\alpha+\beta}\frac{\lambda^*}{K^*} & -\frac{\beta\delta}{\alpha+\beta}\frac{\lambda^*}{E^*} & 0 \end{pmatrix}$$

The eingenvalues of $J(P^*)$ are the roots of the following characteristic polynomial:

$$P_1(z) = z^3 - tr(J)z^2 + Mz - |J|$$

where:

$$tr(J) = \delta + \frac{\beta\Lambda\Omega}{\alpha+\beta} + \overline{E} - 2E^* \tag{S.30}$$

$$M = \delta(\overline{E} - 2E^*) - \frac{\beta\delta}{(\alpha+\beta)K^*\lambda^*} \tag{S.31}$$

$$|J| = -\frac{\beta\delta\Lambda}{(\alpha+\beta)K^*\lambda^*}\left(\Omega + \frac{\overline{E}-2E^*}{\Lambda}\right) \tag{S.32}$$

It is easy to check that the determinant $|J|$ can be expressed as follows:

$$|J| = \frac{\beta\delta}{(\alpha+\beta)K^*\lambda^*}\Lambda\left[g'(E^*) - f'(E^*)\right]$$

where f' and g' are the derivatives of f and g evaluated at E^*. Therefore, $|J| \gtrless 0$ for $\Lambda\left[g'(E^*) - f'(E^*)\right] \gtrless 0$. Since, at the stationary state A, the condition $\Lambda\left[g'(E^*) - f'(E^*)\right] < 0$ holds, A is either a saddle with two eingenvalues with strictly positive real parts or a sink. However, as in the previous section, we can easily exclude the attractivity of A. Notice that $\overline{E} - 2E^* > 0$ always holds in A (see formula (11) of the paper and the definition of the stationary state of type A); this implies that $tr(J) > 0$ if $\Lambda > 0$ (see (S.30)). If $\Lambda < 0$, then $|J| < 0$ if and only if $\Omega\Lambda + \overline{E} - 2E^* > 0$ (see (S.32)), which implies $tr(J) > 0$ (see (S.30)).

At the stationary state B, $|J| > 0$ holds; therefore B is either a saddle with two eingenvalues with strictly negative real parts or a source. [1] finds that a positive determinant and a negative coefficient M are sufficient conditions for saddle-point stability. Notice that, if $\Lambda > 0$, the condition $\overline{E} - 2E^* < 0$ holds (see formula (11) of the paper and the definition of the stationary state of type B) and consequently $M < 0$. In case $\Lambda < 0$, from formula (7) of the paper and the equation $\dot{K}_L = 0$, we obtain:

$$\frac{1}{\lambda^*} = \frac{\alpha+\beta}{\alpha}\delta K^* + (1-\gamma)(\frac{\gamma}{r})^{\frac{\gamma}{1-\gamma}}$$

Substituting in (S.31) and remembering that $K_L^\star < \overline{K}_L = \alpha\delta^{-1}\Gamma^{-\alpha-\beta}$, we can write:

$$M = \delta\left[\overline{E} - 2E^* - \frac{\beta}{\alpha}\delta - \frac{\beta(1-\gamma)(\frac{\gamma}{r})^{\frac{\gamma}{1-\gamma}}}{(\alpha+\beta)K^*}\right] < \delta\left[\overline{E} - 2E^* - \frac{\beta\delta}{\alpha(\alpha+\beta)}\right] \tag{S.33}$$

Therefore, a sufficient condition for saddle-point stability is:

$$E^* > \frac{\overline{E}}{2} - \frac{\beta\delta}{2\alpha(\alpha+\beta)}$$

This completes the proof.

Proof of Proposition 6: Formula (23) follows from (4) while formula (24) follows from (S.10) and

(23). To prove (25), let us remember that $\dot{K}_L = 0$ holds for (see (S.1)):

$$C_L = K_L^{\alpha} E^{\beta} L^{1-\alpha-\beta} + w(1-L) \tag{S.34}$$

and $\dot{\lambda} = 0$ holds for (see (S.2)):

$$\delta = \alpha K_L^{\alpha-1} E^{\beta} L^{1-\alpha-\beta} \tag{S.35}$$

Multiplying both sides of (S.35) by K_L we get $\frac{\delta}{\alpha} K_L = K_L^{\alpha} E^{\beta} L^{1-\alpha-\beta}$; substituting in (S.34) and taking into account formulas (23) and $w = (1-\gamma)\left(\frac{\gamma}{r}\right)^{\frac{\gamma}{1-\gamma}}$, we can write:

$$\begin{aligned} C_L &= \frac{\delta}{\alpha} K_L + (1-\gamma)\left(\frac{\gamma}{r}\right)^{\frac{\gamma}{1-\gamma}} (1-L) = \\ &= \frac{\delta}{\alpha} K_L + \frac{r(1-\gamma)}{\gamma} K_I \end{aligned}$$

Finally, formula (25) is obtained by substituting (23) in (24):

$$C_L^* = (1-\gamma)\left(\frac{\gamma}{r}\right)^{\frac{\gamma}{1-\gamma}} + \frac{\delta}{\alpha}\left[1 - (1-\gamma)\left(\frac{\gamma}{r}\right)^{\frac{\gamma}{1-\gamma}} \Gamma^{\alpha+\beta}\right] K_L^* = (1-\gamma)\left(\frac{\gamma}{r}\right)^{\frac{\gamma}{1-\gamma}} + \frac{\delta(\alpha+\beta)}{\alpha} K_L^*$$

Proofs of comparative statics

Below the straight line $K_L = \overline{K}_L$, the stationary states (without specialization) are given by the intersections between the two following curves:

$$K_L = f(E) := \Omega E \tag{S.36}$$

$$K_L = g(E) := \frac{\eta \left(\frac{\gamma}{r}\right)^{\frac{\gamma}{1-\gamma}} - E(\overline{E} - E)}{\Lambda} \tag{S.37}$$

where:

$$\Omega := \left(\alpha \delta^{-1} \Gamma^{1-\alpha-\beta}\right)^{\frac{\alpha+\beta}{\beta}} \tag{S.38}$$

$$\Lambda := \frac{\delta\left[\eta(1-\alpha-\beta) - \epsilon(1-\gamma)\right]}{\alpha(1-\gamma)} \tag{S.39}$$

Notice that $\Omega > 0$ always holds while:

$$\Lambda > 0 \quad if \quad \epsilon < \epsilon_\Lambda := \eta \frac{1-\alpha-\beta}{1-\gamma} \tag{S.40}$$

Let us rewrite equations (S.36) and (S.37) as follows:

$$F(K_L, E) = K_L - \Omega E = 0 \tag{S.41}$$

$$G(K_L, E) = K_L - \frac{\eta \left(\frac{\gamma}{r}\right)^{\frac{\gamma}{1-\gamma}} - E(\overline{E} - E)}{\Lambda} = 0 \tag{S.42}$$

Differentiating equations (S.41) and (S.42) with respect to the parameter $y = \overline{E}$, ϵ, η, r we obtain:

$$\begin{pmatrix} 1 & -\Omega \\ 1 & \frac{\overline{E} - 2E}{\Lambda} \end{pmatrix} \begin{pmatrix} \frac{\partial K_L}{\partial y} \\ \frac{\partial E}{\partial y} \end{pmatrix} = \begin{pmatrix} \frac{\partial F}{\partial y} \\ \frac{\partial G}{\partial y} \end{pmatrix} \tag{S.43}$$

a) **Proof of Proposition 8:** Posing $y = \overline{E}$, the system (S.43) becomes:

$$\frac{\partial K_L}{\partial \overline{E}} = \Omega \frac{\partial E}{\partial \overline{E}}$$
$$\frac{\partial K_L}{\partial \overline{E}} + \frac{\overline{E} - 2E}{\Lambda} \frac{\partial E}{\partial \overline{E}} = -\frac{E}{\Lambda}$$

Therefore $sign\left(\frac{\partial K_L}{\partial \overline{E}}\right) = sign\left(\frac{\partial E}{\partial \overline{E}}\right)$ and $\frac{\partial E}{\partial \overline{E}} = \frac{E}{\Lambda\left[g'(E) - f'(E)\right]} > 0$ (remember that $\Lambda\left[g'(E) - f'(E)\right] > 0$ at the stationary state B). The part of the Proposition 8 concerning the effects of an increase in $\overline{E}$ (and, consequently, in K_L) on the values of L, C_L and K_I can be easily checked by applying Proposition 6 of the paper.

b) **Proof of Proposition 9:** Posing $y = \eta$, the system (S.43) becomes:

$$\frac{\partial K_L}{\partial \eta} = \Omega \frac{\partial E}{\partial \eta}$$
$$\frac{\partial K_L}{\partial \eta} + \frac{\overline{E} - 2E}{\Lambda} \frac{\partial E}{\partial \eta} = \frac{\delta}{\alpha \Lambda^2} \left[\frac{1-\alpha-\beta}{1-\gamma} E(\overline{E} - E) - \epsilon \left(\frac{\gamma}{r}\right)^{\frac{\gamma}{1-\gamma}} \right]$$

Therefore:

$$sign\left(\frac{\partial K_L}{\partial \eta}\right) = sign\left(\frac{\partial E}{\partial \eta}\right) \tag{S.44}$$

and

$$\frac{\partial E}{\partial \eta} = \frac{\frac{\delta}{\alpha\Lambda^2}\left[\frac{1-\alpha-\beta}{1-\gamma}E(\overline{E}-E) - \epsilon\left(\frac{\gamma}{r}\right)^{\frac{\gamma}{1-\gamma}}\right]}{f'(E)-g'(E)} \tag{S.45}$$

which, substituting $E(\overline{E}-E) = \eta\left(\frac{\gamma}{r}\right)^{\frac{\gamma}{1-\gamma}} - \Omega\Lambda E$ (equation obtained by equalizing the right sides of formulas (S.36) and (S.37)), can be rewritten as follows:

$$\frac{\partial E}{\partial \eta} = \frac{\frac{\delta}{\alpha\Lambda}\left[\frac{\delta}{\alpha}\left(\frac{\gamma}{r}\right)^{\frac{\gamma}{1-\gamma}} - \frac{1-\alpha-\beta}{1-\gamma}\Omega E\right]}{f'(E)-g'(E)}$$

Being $K_L = \Omega E < \overline{K}_L$, where $\overline{K}_L = \frac{\delta(1-\gamma)}{\alpha(1-\alpha-\beta)}\left(\frac{\gamma}{r}\right)^{\frac{\gamma}{1-\gamma}}$ (see formula (9) of the paper), the expression in square brackets is strictly positive; therefore $\frac{\partial E}{\partial \eta} < 0$; this implies, by (S.44), that $\frac{\partial K_L}{\partial \eta} < 0$ holds. The part of the proposition concerning the effects of an increase in η on the values of L, C_L and K_I follows from Proposition 6. The comparative statics results about the parameter ϵ can be easily checked by following the same steps.

c) **Proof of Proposition 10:** For simplicity, we define $\Theta := \eta\left(\frac{\gamma}{r}\right)^{\frac{\gamma}{1-\gamma}}$. Posing $y = r$, the system (S.43) becomes:

$$\begin{aligned} \frac{\partial K_L}{\partial r} - \Omega\frac{\partial E}{\partial r} &= \frac{\partial \Omega}{\partial r}E \\ \frac{\partial K_L}{\partial r} + \frac{\overline{E}-2E}{\Lambda}\frac{\partial E}{\partial r} &= \frac{1}{\Lambda}\frac{\partial \Theta}{\partial r} \end{aligned}$$

where:

$$\frac{\partial \Theta}{\partial r} = -\frac{\gamma}{r(1-\gamma)}\Theta < 0 \tag{S.46}$$

$$\frac{\partial \Omega}{\partial r} = \frac{\gamma(1-\alpha-\beta)}{\beta r(1-\gamma)}\Omega > 0 \tag{S.47}$$

The solution of such system is:

$$\frac{\partial K_L}{\partial r} = \frac{\partial \Omega}{\partial r}E + \frac{\partial E}{\partial r}\Omega \tag{S.48}$$

$$\frac{\partial E}{\partial r} = \frac{\frac{1}{\Lambda}\frac{\partial \Theta}{\partial r} - \frac{\partial \Omega}{\partial r}E}{f'(E) - g'(E)} \tag{S.49}$$

Notice that, if $\Lambda > 0$, then $\frac{\partial E}{\partial r} > 0$ always holds[3]. If $\Lambda < 0$, then $\frac{\partial E}{\partial r} > 0$ holds if and only if:

$$E < \frac{\frac{1}{\Lambda}\frac{\partial \Theta}{\partial r}}{\frac{\partial \Omega}{\partial r}} \tag{S.50}$$

Substituting formulas (S.46) and (S.47) in (S.50), the inequality (S.50) can be expressed as:

$$E < \frac{\alpha\beta(1-\gamma)\Theta}{\delta\Omega(1-\alpha-\beta)\left[\epsilon(1-\gamma) - \eta(1-\alpha-\beta)\right]}$$

Remembering that, in B, $K_L = \Omega E < \overline{K}_L = \frac{\delta(1-\gamma)}{\alpha(1-\alpha-\beta)}\left(\frac{\gamma}{r}\right)^{\frac{\gamma}{1-\gamma}}$ holds (see (S.41) and formula (9) of the paper) and solving the inequality:

$$\frac{\overline{K}_L}{\Omega} < \frac{\alpha\beta(1-\gamma)\Theta}{\delta\Omega(1-\alpha-\beta)(\epsilon(1-\gamma) - \eta(1-\alpha-\beta))}$$

we obtain the sufficient condition for $\frac{\partial E^*}{\partial r} > 0$ given in the proposition.

The sufficient condition for $\frac{\partial E^*}{\partial r} < 0$ is obtained by following similar steps. Remember first that for $\overline{E} = \overline{E}_1$ the curves $f(E)$ and $g(E)$ are tangent; it is easy to check that, at the tangency point, $E = \sqrt{\Theta}$ holds. This implies that, for $\overline{E} > \overline{E}_1$, $E > \sqrt{\Theta}$ holds at the stationary state B. Therefore, the sufficient condition for $\frac{\partial E^*}{\partial r} < 0$ is obtained solving the following inequality[4]:

$$\sqrt{\Theta} \geq \frac{\frac{1}{\Lambda}\frac{\partial \Theta}{\partial r}}{\frac{\partial \Omega}{\partial r}}$$

which can be rewritten as:

$$\eta + \frac{\alpha\beta(1-\gamma)\sqrt{\Theta}}{\delta\Omega(1-\alpha-\beta)^2}\sqrt{\eta} - \frac{\epsilon(1-\gamma)}{1-\alpha-\beta} \leq 0$$

[3]Remember that, at the stationary state B, $sign\,(\Lambda) = sign\,[g'(E^*) - f'(E^*)]$ holds.

[4]$\frac{\partial E}{\partial r} < 0$ holds if and only if the opposite of condition (S.50) is satisfied.

Let us now consider the variations in K_L, L and K_I generated by an increase in r. Remember that, according to Proposition 6, K_L is positively correlated with L and negatively correlated with K_I. Consequently, we have only to analyze the sign of $\frac{\partial K_L}{\partial r}$. Notice that, by (S.48), $\frac{\partial E}{\partial r} > 0$ implies $\frac{\partial K_L}{\partial r} > 0$; therefore, if $\Lambda > 0$, then $\frac{\partial K_L}{\partial r} > 0$. If $\Lambda < 0$, the inequality $\frac{\partial K_L}{\partial r} > 0$ can be written as follows (we use formulas (S.48), (S.46) and (S.47)):

$$\frac{\partial K_L}{\partial r} = \frac{\gamma}{r(1-\gamma)} \frac{\Omega}{\Lambda} \left[\frac{1-\alpha-\beta}{\beta} E \left(\overline{E} - 2E \right) - \Theta \right]$$

Therefore, $\frac{\partial K_L}{\partial r} > 0$ if and only if:

$$\frac{1-\alpha-\beta}{\beta} E(\overline{E} - 2E) - \Theta < 0.$$

References

1. Wirl F (1997) Stability and limit cycles in one-dimensional dynamic optimisations of competitive agents with a market externality. Journal of Evolutionary Economics 7:73-89.
